# Supplementary material for: Dielectric Elastomers UV-Cured from Poly(dimethylsiloxane) Solution in Vinyl Acetate
Source: Polymers (Basel). 2020 Nov 11;12(11):2660. doi: 10.3390/polym12112660 (PMC7697987; doi:10.3390/polym12112660)
Supplement: Supplementary file 1 [file polymers-12-02660-s001.pdf]

Supporting Information

Dielectric Elastomers UV-Cured from Poly(dimethylsiloxane) Solution in Vinyl Acetate

Seung Koo Park*, Meejeong Choi, Dong Wook Kim, Bong Je Park, Eun Jin Shin, Suntak Park*, Sungryul Yun*

*Correspondence to skpark@etri.re.kr, spark@etri.re.kr, sungryul@etri.re.kr

Experimantal section

Materials: Diethoxydimethylsilane (**1**) and diethoxymethylvinylsilane (**2**) were purchased from Tokyo Chemical Industry Co., Ltd. Vinyl acetate (VAc), a platinum (0)-1,3-divinyl-1,1,3,3,-tetramethyldisiloxane complex solution (0.05 mol% of platinum), a 37 % hydrochloric solution in water, and chloroform-d_1 were purchased from Aldrich Chemical Co., Inc. Ethyl acetate (EA), sodium hydroxide, sodium sulfate, and anhydrous magnesium sulfate were purchased from Junsei Chemical Co., Ltd. 1-Hydroxycyclohexyl phenyl ketone was obtained from Ciba Specialty Chemicals Inc. All reagents were used as received.

Measurements: A Shimadzu UV-2600 spectrometer and a Nicolet 6700 FT-IR spectrometer were used for UV-vis and IR measurements, respectively. VPDMS or the VPDMS solution in VAc was coated on a NaCl window for the IR measurement. In the case of the VPDMS solution, the layer on the NaCl window was directly converted to the UV-cured film in the same way as the film was prepared before. An attenuated total reflection (ATR) technique was used in order to compare IR spectroscopy results before and after sopinification of the UV-cured film. A monolithic diamond crystal is mounted on the ATR accessory. ^{1}H and ^{13}C NMR spectra were recorded with a Bruker 500 MHz NMR spectrometer. Chloroform-d_1 was used as a solvent for the NMR measurements. Mechanical properties of the films were measured by using TA Instrument RSA-G2. The films prepared from the VPDMS

solution were cut into a 5 × 30 mm strip. Initial length of the sample film was 10 mm. Five sample films were taken for each film and the samples with highest and lowest strain were excluded from the data analysis. The extension rate was 0.1 mm/s. Electrical properties of the films were obtained by using TA Instrument RSA-G2 and analyzed with an Agilent E4890A Precision LCR meter. The electrode was formed 20 mm in diameter on both side of the film by vacuum-deposition of gold. Spectrum intensities of the UV lamp were measured with an UV Power Puck® II radiometer.

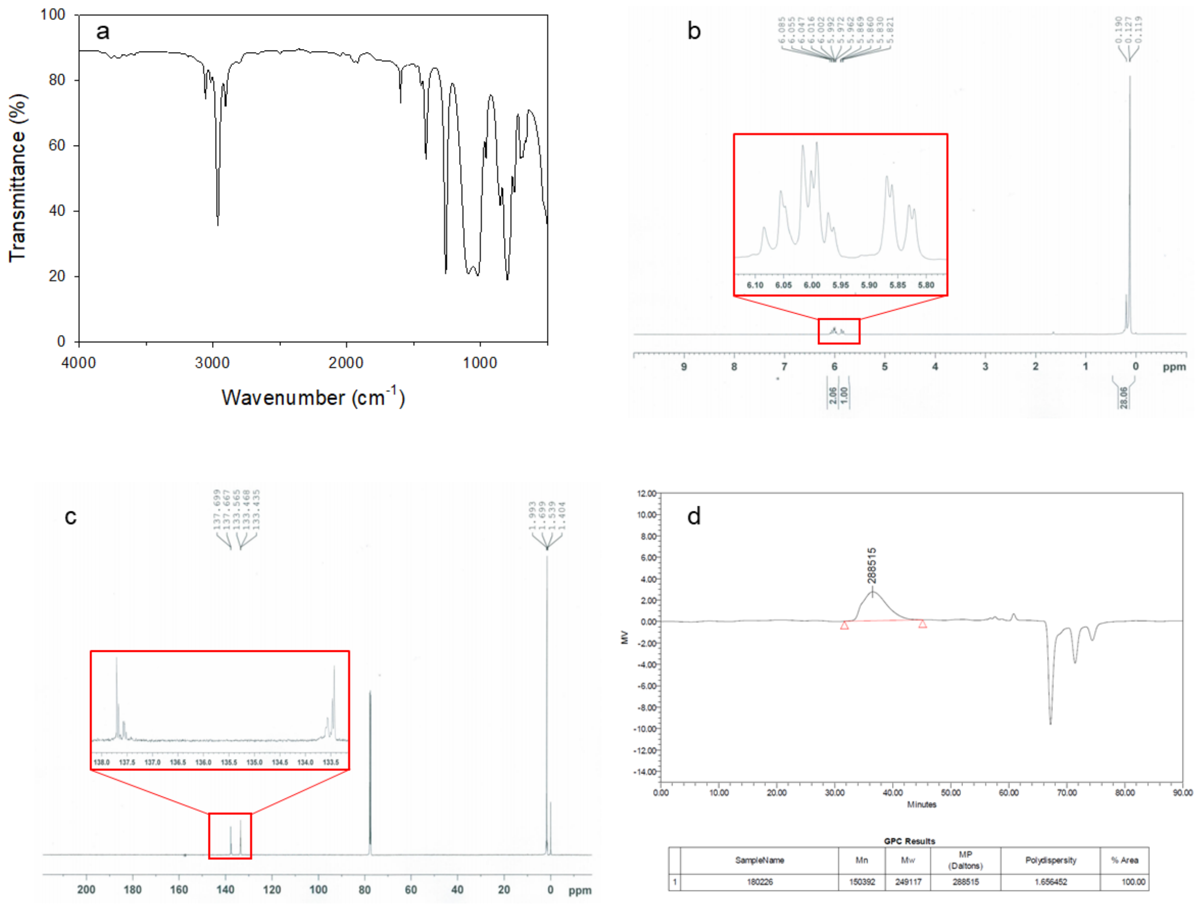

Figure S1. IR (a), ^{1}H-NMR (b), ^{13}C-NMR (c), and GPC (d) spectra of VPDMS.

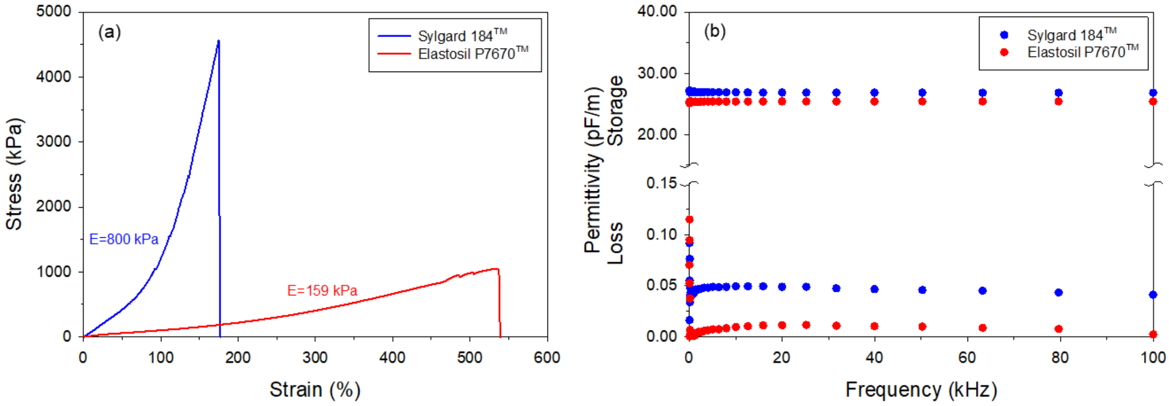

Figure S2. Stress-strain curves (a) and dielectric permittivity (b) of the PDMS films prepared from Sylgard 184™ and Elastosil P7670™.

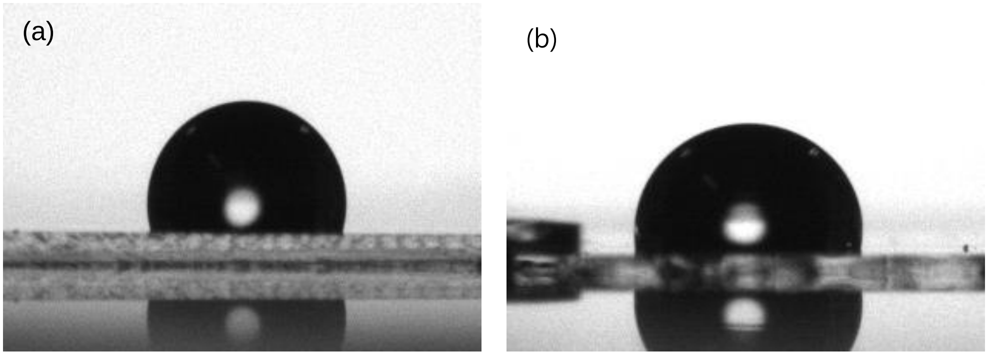

Figure S3. Water contact angles of the UV-cured film prepared from the V10 solution before (a) and after (b) saponification.

Table S1. UV lamp intensity applied on the solution layers.

Spectra	UV-V	UV-A	UV-B	UV-C
Intensity (mW/cm^2)	7.1	8.3	5.8	2.5

UV-V: 395~445 nm; UV-A: 320~390 nm; UV-B: 280~320 nm; UV-C: 250~260 nm (From a manual of the radiometer).
